# Stroke Outcome Measurements From Electronic Medical Records: Cross-sectional Study on the Effectiveness of Neural and Nonneural Classifiers

**DOI:** 10.2196/29120

**Published:** 2021-11-01

**Authors:** Bruna Stella Zanotto, Ana Paula Beck da Silva Etges, Avner dal Bosco, Eduardo Gabriel Cortes, Renata Ruschel, Ana Claudia De Souza, Claudio M V Andrade, Felipe Viegas, Sergio Canuto, Washington Luiz, Sheila Ouriques Martins, Renata Vieira, Carisi Polanczyk, Marcos André Gonçalves

**Affiliations:** 1 National Institute of Health Technology Assessment - INCT/IATS (CNPQ 465518/2014-1) Universidade Federal do Rio Grande do Sul Porto Alegre Brazil; 2 Graduate Program in Epidemiology Universidade Federal do Rio Grande do Sul Porto Alegre Brazil; 3 School of Technology Pontifícia Universidade Católica do Rio Grande do Sul Porto Alegre Brazil; 4 Graduate Program of Computer Science Universidade Federal do Rio Grande do Sul Porto Alegre Brazil; 5 Brazilian Stroke Network Hospital Moinhos de Vento Porto Alegre Brazil; 6 Computer Science Department Universidade Federal de Minas Gerais Belo Horizonte Brazil; 7 Centro Interdisciplinar de História, Culturas e Sociedades (CIDEHUS) Universidade de Évora Évora Portugal

**Keywords:** natural language processing, stroke, outcomes, electronic medical records, EHR, electronic health records, text processing, data mining, text classification, patient outcomes

## Abstract

**Background:**

With the rapid adoption of electronic medical records (EMRs), there is an ever-increasing opportunity to collect data and extract knowledge from EMRs to support patient-centered stroke management.

**Objective:**

This study aims to compare the effectiveness of state-of-the-art automatic text classification methods in classifying data to support the prediction of clinical patient outcomes and the extraction of patient characteristics from EMRs.

**Methods:**

Our study addressed the computational problems of information extraction and automatic text classification. We identified essential tasks to be considered in an ischemic stroke value-based program. The 30 selected tasks were classified (manually labeled by specialists) according to the following value agenda: tier 1 (achieved health care status), tier 2 (recovery process), care related (clinical management and risk scores), and baseline characteristics. The analyzed data set was retrospectively extracted from the EMRs of patients with stroke from a private Brazilian hospital between 2018 and 2019. A total of 44,206 sentences from free-text medical records in Portuguese were used to train and develop 10 supervised computational machine learning methods, including state-of-the-art neural and nonneural methods, along with ontological rules. As an experimental protocol, we used a 5-fold cross-validation procedure repeated 6 times, along with *subject-wise sampling*. A heatmap was used to display comparative result analyses according to the best algorithmic effectiveness (F1 score), supported by statistical significance tests. A feature importance analysis was conducted to provide insights into the results.

**Results:**

The top-performing models were support vector machines trained with lexical and semantic textual features, showing the importance of dealing with noise in EMR textual representations. The support vector machine models produced statistically superior results in 71% (17/24) of tasks, with an F1 score >80% regarding care-related tasks (patient treatment location, fall risk, thrombolytic therapy, and pressure ulcer risk), the process of recovery (ability to feed orally or ambulate and communicate), health care status achieved (mortality), and baseline characteristics (diabetes, obesity, dyslipidemia, and smoking status). Neural methods were largely outperformed by more traditional nonneural methods, given the characteristics of the data set. Ontological rules were also effective in tasks such as baseline characteristics (alcoholism, atrial fibrillation, and coronary artery disease) and the Rankin scale. The complementarity in effectiveness among models suggests that a combination of models could enhance the results and cover more tasks in the future.

**Conclusions:**

Advances in information technology capacity are essential for scalability and agility in measuring health status outcomes. This study allowed us to measure effectiveness and identify opportunities for automating the classification of outcomes of specific tasks related to clinical conditions of stroke victims, and thus ultimately assess the possibility of proactively using these machine learning techniques in real-world situations.

## Introduction

### Background

Stroke is the second leading cause of mortality and disability-adjusted life years globally [1,2]. The outcomes of stroke can vary greatly, and timely assessment is essential for optimal management. As such, there has been an increasing interest in the use of automated machine learning (ML) techniques to track stroke outcomes, with the hope that such methods could make use of large, routinely collected data sets and deliver accurate, personalized prognoses [3]. However, studies applying ML methods to stroke, although published regularly, have focused mostly on stroke imaging applications [4-6] and structured data retrieval [3]. Few studies have addressed the unstructured textual portion of electronic medical records (EMRs) as the primary source of information.

Indeed, the use of EMR data in the last decade has led to promising findings in population health research, such as patient-use stratification [7], treatment-effectiveness evaluation [8], early detection of diseases [9], and predictive modeling [10]. However, dealing with EMR data is often labor intensive [11] and challenging because of the lack of standardization in data entry, changes in coding procedures over time, and the impact of missing information [9,12-14]. The information technology (IT) gap between automated data collection from EMRs and improving the quality of care has been described in the literature as a decelerator of value initiatives [15-18].

With recent advances in IT, several groups have attempted to apply natural language processing (NLP) to the text analysis of EMRs to achieve early diagnosis of multiple conditions, such as peripheral arterial disease [19], asthma [20], multiple sclerosis [21], and heart failure [22]. In these studies, NLP was used to find specific words or phrases in a predefined dictionary that described the symptoms or signs of each disease [14,21,23].

### Objectives

Generating value for the patient as the central guide requires advances in strategies to automate the capturing of data that will allow managers to assess the quality of service delivery to patients [24,25]. Accordingly, our research aims to compare the effectiveness of state-of-the-art automatic text classification methods in classifying data to support the prediction of clinical patient outcomes and the extraction of patient characteristics from EMR sentences. With stroke as our case study application, our specific goal is to investigate the capability of these methods to automatically identify, with reasonable effectiveness, the outcomes and clinical characteristics of patients from EMRs that may be considered in a stroke outcome measurement program.

## Methods

### Overview

**Figure 1:**
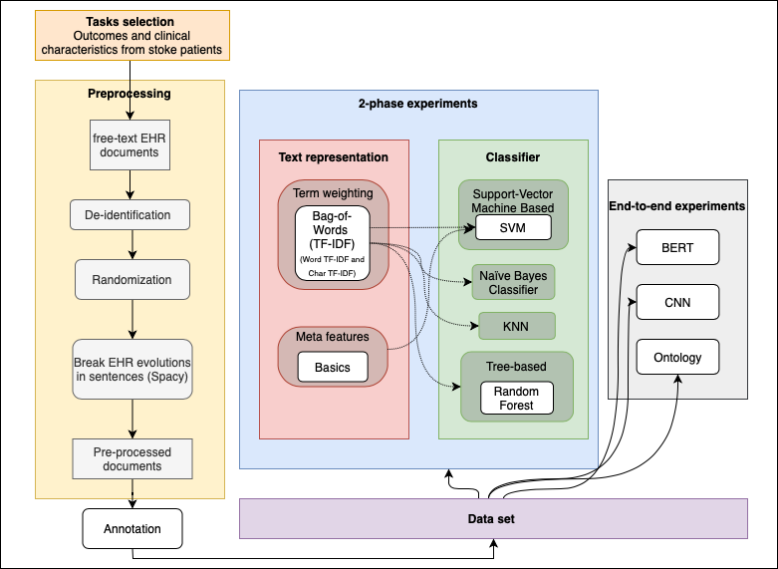
Study architecture. BERT: bidirectional encoder representation from transformers; CNN: convolutional neural network; EHR: electronic health record; KNN: K-nearest neighbor; SVM: support vector machine; TF-IDF: term frequency-inverted document frequency.

This study faced a computational problem related to information extraction and free-text classification. As presented in Figure 1, the dotted lines represent the union of the text representative technique that was used with each classifier in the two-phase experiments. Our study was generally organized into four stages: (1) task selection; (2) study design, preprocessing, and data annotation; (3) definition of automatic text classification methods; and (4) experimental evaluation (experimental protocol, setup, and analysis of results).

### Task Selection

A literature review and multidisciplinary expert interviews (n=8) were used to define specific outcome dimensions and measures that may be considered in an outcome measurement program for ischemic stroke. The outcome identification step was based on adhering to value agenda element dimensions to cover the tiers of the outcome hierarchy [26], such as functionality dimensions, the recovery process, and outcomes that matter to patients. These dimensions included risk events, achieved health care status, and stroke outcome scales, such as the National Institutes of Health Stroke Scale (NIHSS) and the modified Rankin scale (mRS) [27,28].

### Study Design and Data Annotation

We retrospectively built a database of medical records from a digital hospital system. The database covered 2 years of patients hospitalized for ischemic stroke. The hospital is a private institution of excellence in southern Brazil. The EMR system used was the MV Soul (Recife). Since 2017, the hospital has introduced the ICHOM standard sets’ data collection routine for different clinical pathways and created an office for institutional values. To examine the stroke pathway, data were collected on October 15, 2015. In 2019, the hospital incorporated the Angel Awards Program [29], which was certified as a platinum category at the end of the first year. This study was approved by the hospital ethics committee (CAAE: 29694720000005330).

Medical records of patients were submitted to preprocessing using the spaCy Python library (Python Software Foundation; Python Language Reference, version 2.7) [30] to stratify texts into sentences. A total of 44,206 EMR sentences were obtained from 188 patients. The approach followed a hypothesis for managing unbalanced data, such as electronic health records, which assumes that relevant information to be retrieved from EMRs encompasses a small space of words delimited as sentences, and the residual is noise [31-33]. During the text stratification process, spaCy [30] uses rule-based algorithms that set the sentence limits according to the patterns of characters, thereby delimiting its beginning and end. The names of patients and medical staff were identified, thus removing all confidential information from the data set. The preprocessed textual sentence was represented in a vector of words that disregarded grammar and word order but maintained their multiplicity.

For sentence annotation (intratask class labeling), we developed annotation guidelines that provided an explicit definition of each task, its classes (response options), and examples to be identified in the documents. This guideline is written in Portuguese and is available upon request.

Two annotators independently reviewed the preprocessed text documents (44,206 sentences) and had the percent agreement between them measured by κ, which was higher than 0.61 (substantial agreement) [34]. Task-level disagreements were resolved by consensus determination by 2 annotators, with assistance from a committee composed of experts (APE, ACS, MP, KBR, and CAP).

Each task could have two or more output answers, depending on the meaning of the sentence. Examples of an EMR and the annotation process can be seen in Multimedia Appendices 1 and 2. Task details in terms of class and sentence distribution are shown in Multimedia Appendix 3 and demonstrate the highly imbalanced nature of the tasks with most of the sentences belonging to the NI (noninformative) class. This makes it a very hard endeavor from an ML perspective. Subsequently, we evaluated the impact of this imbalance in the experimental results.

### Automatic Text Classification Methods

As presented in the study design, the ML methods were divided into two categories: two-phase methods and end-to-end (E2E) methods [35]. The first category of methods consisted of approaches whose document (ie, sentence) representation was intrinsically independent of the classification algorithm used to predict the class. In other words, the classifier used to predict the class of documents was not used in the construction phase of the document representation. In terms of text representations, we considered three alternatives, namely traditional term-weighting alternatives (term frequency-inverted document frequency [TFIDF]); weighting based on word and character (n-gram) frequency; and recent representations based on meta-features, which capture statistical information from a document’s neighborhood and have obtained state-of-the-art effectiveness in recent benchmarks [35-39].

As two-phase classification algorithms, we exploited support vector machines (SVMs), which are still considered the most robust nonneural network text classification algorithm [35,39,40], random forests (RF), K-nearest neighbor (KNN), and naïve Bayes classifier (NBC), to address the most popular algorithms in terms of classification and retrieval of text information [41-44].

In contrast, E2E methods use a discriminative classifier function to transform the document representation space into a new and more informed (usually more reduced and compact) space and use this classifier to predict the document class. In general, these approaches use an iterative process of representation, classification, evaluation, and parameter adaptation (eg, transform, predict, evaluate loss function, and backpropagate, respectively). For E2E classifiers, we exploited two neural architectures, namely convolutional neural networks (CNNs), which exploit textual patterns such as word co-occurrences, and bidirectional encoder representation from transformers (BERT), which exploits attention mechanisms and constitute the current state-of-the-art in many NLP tasks.

Finally, we exploited a rule-based classifier specialized for the tasks at hand (stroke tasks, represented in the ontology web language [OWL]). The rule-based knowledge model was developed using logical conditions built alongside domain specialists [45]. This technique has shown effectiveness equivalent to that of some ML classification models in certain domains without the need for a large amount of data and training time, which are commonly required by supervised methods [46-49]. In contrast, it is heavily dependent on the specialists and the coverage of the rules on the text expressions. More details about each of the exploited algorithms are provided in Multimedia Appendix 4 [3,35,37,39,41-45,50-63].

The two-phase methods used in this research are referred to as the representation technique combined with the classification algorithm, as follows: word-TFIDF and character-TFIDF combined with SVM (SVM+W+C), Bag-of-Words (BoW) combined with SVM (SVM+BoW), meta-features combined with SVM (meta-features), word-TFIDF combined with SVM (SVM+Word-TFIDF), character-TFIDF combined with SVM (SVM+Chard-TFIDF), Word-TFIDF combined with random forest (RF+Word-TFIDF), word-TFIDF combined with KNN (KNN+Word-TFIDF), and word-TFIDF combined with naïve Bayes (Naïve Bayes+Word-TFIDF). In contrast to TFIDF, BoW explores only the frequency of terms (term frequency) and not the frequency of terms in the collection (IDF component). The E2E methods are simply called CNN and BERT, and the ontological method is called OWL.

### Experimental Evaluation

#### Overview

The experimental process consisted of testing different classification methods with sets of annotated data to assess and compare their performances (effectiveness). The experimental procedure, described in Multimedia Appendix 5, consisted of four phases: (1) representing the free-text sentences as numerical vectors, (2) the training and tuning process (in a validation set) by means of a folded cross-validation procedure, (3) the execution of the classification algorithms in the test set and effectiveness assessment, and (4) the synthesis of the results in a heatmap table.

A classification model was developed for each task. Each task resulted in an individual automatic classification model for the training and testing process of the model. As an experimental protocol, we used a five-fold cross-validation procedure repeated six times (resulting in 30 test samples). We also exploited *subject-wise cross-validation* in the sense that the information from the same patient was always assigned to the same fold to test the ability of the model to predict new data that was not used in the learning process. These procedures address potential problems, such as overfitting and selection bias [64], and produce results that are more reliable.

To evaluate the ability to classify the relevant Brazilian-Portuguese medical free-text records correctly, we used the Macro-F1 score (equation 1). This metric is based on a *confusion matrix* and is defined as follows:



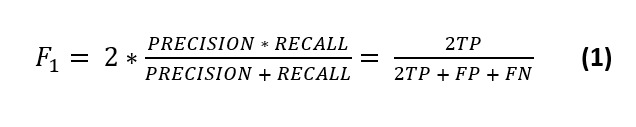



where TP is true positive, TN is true negative, FP is false positive, and FN is false negative. Precision (positive predictive value) = TP / TP + FP = the number of returned hits that were true positive. Recall (sensibility) = TP / TP + FN = is the fraction of the total number of true positives retrieved.

The F1 measure is calculated for each class. Macro-F1 summarizes the classification effectiveness by averaging F1 values for all classes. Macro-F1 is one of the most popular aggregated evaluation metrics for the classifier evaluation of unbalanced or skewed data sets [42,65,66]. Macro-F1 is especially suitable for imbalanced data sets, as the effectiveness of each individual class contributes equally to producing a final score. For instance, in a task with four classes, in which one of them is NI, if all classes are predicted as NI, the Macro-F1 score will be no higher than 0.25 (F1 of 1 for NI and 0 for the three other classes). Accuracy or any other evaluation measure focused on the instance, instead of the class effectiveness, would produce a very high score (close to 1 in this particular case).

To compare the average results of our cross-validation experiments, we assessed statistical significance by using a paired two-tailed *t* test with 95% CIs. To account for multiple tests, we adopted the Friedman-Nemenyi test [67] with Bonferroni correction for multiple comparisons of mean rank sums. The Friedman test was used to compare multiple methods.

We consider that making the data and the code used in our experimental protocol available to others is potentially useful for reproducibility and for use in other studies. Both the code and data will be available upon request. The mood-specific parameter tuning details are presented in Multimedia Appendix 6.

#### Experimental Analysis

The experiments aimed to provide relationships between the classification methods and the tasks, allowing for connecting the best methods with each outcome measure or patient characteristics. Considering that the model’s results can influence health decision-making in some way, the F1 score thresholds may vary depending on the type of class and the imbalance of the data. We reported the results by means of a heatmap, adopting a red color for F1<20%, a gradual color scale from orange to yellow for 21%<F1<79%, and green for F1>80% [68-71]. Tasks (represented by the lines) were ordered by the average of the performed models, whereas the ordering of the columns shows the rank position of each method according to the statistical analysis.

For the sake of the fairness of the comparison, the OWL technique should not be and is not directly compared and ranked herein along with the other ML models described above that require a combination of text representations with trained classification algorithms. OWL rules were designed to work with the entire corpus (including the test) and were not designed for generalization. Instead, they are built to work well in the specific domain or task for which they were created. In any case, for reasons of practical application and as a research exercise, as a secondary analysis, we compared (later) the OWL technique with the ML model ranked as the best based on the Friedman test. This analysis allowed us to identify the weaknesses and strengths of both approaches (generalized ML models vs domain or task-specific ontological rules) in the contrasting tasks.

Moreover, we performed a feature selection analysis [72,73]. This technique is used to rank the most informative features of each task according to the information theory criteria. In particular, we used SelectKBest (Python Software Foundation; Python Language Reference, version 2.7) with the chi-square, which is independent of the classification algorithms used [74]. This final analysis helps in understanding how ML can help with outcome measurements for the stroke care pathway, potentially boosting advances in quality indicator automation.

Finally, to complete the analysis and evaluate the impact of the highly skewed distribution, especially toward the NI class, we ran an experiment in which we performed a random undersampling process for all considered tasks (we used the RandomUnderSampler Phyton library [75]). In detail, we randomly selected the same number of training random examples of the NI as the number of instances of the second largest (non-NI) class of a given task. We then reran all ML classifiers (the ontology method is not affected by this process as it has no training) in all 24 tasks, considering as the training set the reduced (undersampled) NI training samples along with the same (unchanged) previous samples for the other classes. We did that for all six rounds of five-fold cross-validation of our experimental procedure, changing the seed for selection in each round, resulting in six different NI reduced training sets. The test folds in all cases remain unchanged, meaning that we keep the same skewed distribution as in the original data set, as we do not know the class of the test instances.

## Results

### Tasks Selection

Discussions with experts in the stroke care pathway allowed us to define 30 tasks that were considered feasible to extract from EMRs. For the first tier, the standard sets were usually defined to evaluate the clinical stroke outcomes that were used, including the mRS [27] and the NIHSS scales [76], in addition to traditional outcomes such as mortality and pain level. For tier 2, the ICHOM standard set developed for ischemic stroke was used [77], which considers measures of mobility, ability to communicate, ability to feed orally, the ability to understand, and measures and scales of strength level. Indicators of the hospitalization care process used in the institution were also included, such as rating scales and risk events tracked by fall risk, pressure ulcer risk, fall events during hospitalization, infection indicators, intracranial hemorrhage, therapy care (thrombolytic, thrombectomy, or both), and the location of the patient during the inpatient path [78]. Finally, baseline characteristics important for tracking the population and further risk-adjusted analysis were included [79], such as high blood pressure, smoking status, coronary artery disease, atrial fibrillation, diabetes, prior stroke, active cancer, alcoholism, obesity, and dyslipidemia. Each category, containing the tasks and their respective classes, is presented in Table 1.

**Table 1:** Eligible tasks for analysis and classification rules.

Tasks		Number of classes	Supporting information for classes
**Health care status achieved (tier 1)**			
	Rankin	8	0-6 NI^a^
	National Institutes of Health Stroke Scale	42	1-41 NI
	Death	3	Absence of vital signs Vital signs present NI
**Process of recovery (tier 2)**			
	Mobility level	16	1-15 NI
	Self-care	3	Able Unable NI
	Pain	4	No pain Low to intermediate pain Intense pain NI
	Strength	7	0-5 NI
	Paresis	3	Yes No NI
	Ability to feed orally	3	Yes No NI
	Ability to communicate	4	Yes No Poorly or symptomatic NI
	Ability of understanding	4	Yes No Poorly or symptomatic NI
	Ability to ambulate	4	Yes No Poorly or symptomatic NI
**Treatment or care related**			
	Thrombolytic therapy	3	No delta Yes NI
	Thrombectomy	3	No delta Yes NI
	Location	4	Emergency room ICU^b^ Inpatient unit NI
	Infection indication	3	Yes No NI
	Intracranial hemorrhage	3	Yes No NI
	Fall risk	4	Low risk Moderate risk High risk NI
	Pressure ulcer risk	4	Low risk Moderate risk High risk NI
	Fall event during inpatient	3	Yes No NI
**Baseline characteristics**			
	High blood pressure	3	Yes No NI
	Smoking status	4	Yes No Former NI
	Coronary artery disease	3	Yes No NI
	Atrial fibrillation	3	Yes No NI
	Diabetes	3	Yes No NI
	Prior stroke	3	Yes No NI
	Cancer	3	Yes No NI
	Alcoholism	4	Yes No Former NI
	Obesity	3	Yes No NI
	Dyslipidemia	3	Yes No NI

^a^NI: noninformative.

^b^ICU: intensive care unit.

After the identification of all tasks and the annotation process, the analysis proceeded only with tasks that had substantial (0.61>κ>0.80) and almost perfect (κ≥0.81) agreement between annotators [34]. A total of six tasks were excluded from the final analysis because of moderate or fair agreement or disagreement: (1) active cancer information, (2) strength level, (3) intracranial hemorrhage, (4) ability to understand, (5) self-care, and (6) fall events during inpatient visits. All documents were labeled by the annotators, and the median κ regarding the 24 remaining tasks was 0.74 (IQR 0.65-0.89; substantial agreement).

### Patient Characteristics

The descriptive characteristics of patients, including previous comorbidities, NIHSS score, and clinical care, are presented in Table 2.

**Table 2:** Descriptive characteristics of the patients.

Characteristics			Patients with ischemic stroke evaluated (n=188)		
			Values, median (range)		Values, n (%)
Age (years)			79 (68-87)		N/A^a^
LOS^b^ (days)			6 (4-12)		N/A
**Sex**					
	Female	N/A		100 (53)	
	Male	N/A		88 (47)	
**Comorbidities**					
	Previous stroke	N/A		38 (20)	
	Previous coronary artery disease	N/A		12 (6)	
	Atrial fibrillation	N/A		33 (18)	
	Diabetes	N/A		53 (28)	
	Hypertension	N/A		125 (66)	
	Smoking status	N/A		15 (8)	
	Alcoholism	N/A		4 (2)	
**Treatment and care related**					
	Antithrombotic therapy	N/A		131 (70)	
	Thrombolysis with rtPA^c^	N/A		38 (20)	
	Thrombectomy	N/A		12 (6)	
	Thrombolysis and thrombectomy	N/A		7(4)	
**NIHSS^d^**					
	<8	N/A		147 (78)	
	>8 and <15	N/A		24 (13)	
	>15	N/A		17 (9)	

^a^N/A: not applicable.

^b^LOS: length of stay.

^c^rtPA: alteplase.

^d^NIHSS: National Institutes of Health Stroke Scale.

### Experimental Results

**Figure 2:**
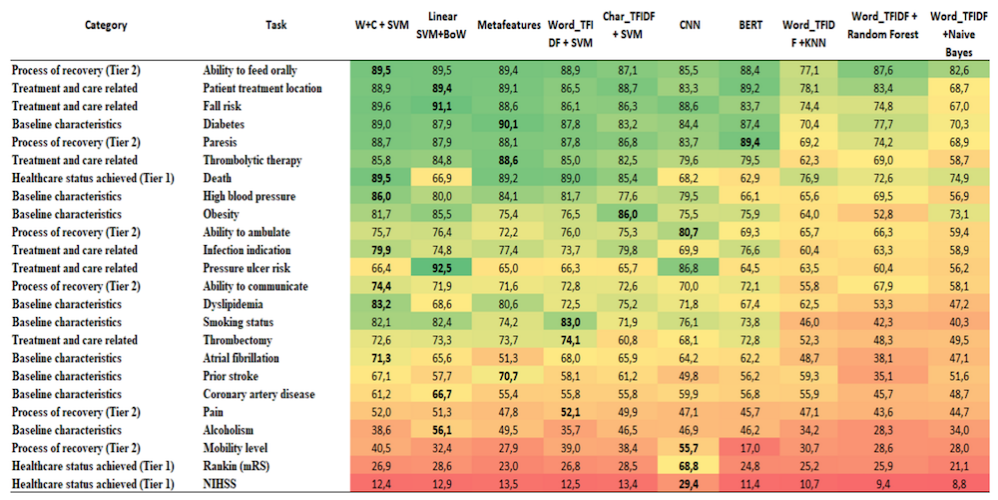
Results of Macro-F1 for each task and comparative models (expressed in percentage). BERT: bidirectional encoder representation from transformers; CNN: convolutional neural network; mRS: Modified Rankin Score; NIHSS: National Institutes of Health Stroke Scale; SVM+BoW: support vector machine plus Bag-of-Words; TFIDF: term frequency-inverted document frequency; W+C+SVM: word-term frequency-inverted document frequency and character-term frequency-inverted document frequency combined with support vector machine.

The Macro-F1 values for each of the 24 tasks using the 10 compared models are shown in Figure 2. Considering each task separately, there is no single method that always dominates, and there is no agreement on a unique category of tasks that perform better. The ML models SVM+W+C and SVM+BoW were the best and most consistent techniques used in this data set. Both techniques use term-weighting representations that are used alongside SVM classifiers. The latter simply exploits within-document word term frequencies (term frequency), whereas the former, in addition to exploiting data set–oriented term statistics (IDF), also builds character-based n-gram representations of the words in the vocabulary. The character-based n-grams, despite increasing the vocabulary size and sparsity, help to deal with misspellings and word variations that are common in EMRs, which might explain the SVM+W+C good results.

The SVM+W+C model excels in tasks belonging to different categories, such as the ability to feed orally (Tier 2: the process of recovery), with an F1 score of 89.5% (95% CI 89.2%-89.8%); death (tier 1: health care status achieved), with an F1 score of 89.5% (95% CI 87.5%-92.5%); and high blood pressure and dyslipidemia (the baseline characteristics of patients), with F1 scores of 86% (95% CI 83.8%-88.2%) and 83.2% (95% CI 77%-89%), respectively. SVM+BoW, in turn, excels in tasks belonging to the treatment- or care-related categories, such as patient location during treatment (F1 score 89.4%; 95% CI 88%-91%), fall risk (F1 score 91.1%; 95% CI 90.1%-92.1%), and pressure ulcer risk (F1 score 92.5; 95% CI 91.5%-93.5%). The meta-features model, which also exploits SVM as a classifier but uses a completely different text representation, was on average, the third-best placed ML model to cover more tasks with good effectiveness, except in tasks such as diabetes (F1 score 90.1%; 95% CI 88.8%-91.4%) and thrombolytic therapy (F1 score 88.6%; 95% CI 87.5%-90.1%), in which it was the sole winner model (best performer with no ties). The models that used SVM but exploited either only word- or character-based representations came in the fourth and fifth places, losing to methods that exploited both representations in a conjugated way.

The neural methods CNN and BERT were grouped in the middle, with only moderate effectiveness in most tasks. This outcome is mostly due to the lack of sufficient training data for the optimal deployment of these methods. Indeed, previous work has demonstrated that neural solutions are not adequate for tasks with low to moderate training data, and they can only outperform other more traditional ML methods in text classification tasks when presented with massive amounts of training [35,39], which is generally uncommon in the health domain.

Regarding the effectiveness of the tasks, patient characteristics and care-related process tasks produced better effectiveness. Five of them are examples of good adherence with multiple models, including patient treatment location, fall risk, thrombolytic therapy, diabetes, and paresis, all with multiple models with high effectiveness. Tasks related to measures of mobility, ability to communicate, ability to ambulate, and pain did not achieve high Macro-F1 values in most models.

The tasks with many classes, such as NIHSS (42 classes), mobility level (n=16), and Rankin (n=8), performed worse, regardless of the model. This outcome is mostly due to issues related to the very skewed distribution (high imbalance) found in our unstructured real-life data set. Indeed, the high percentage of NI in the document penalizes effectiveness, mainly for the minor classes, which are captured more faithfully by the Macro-F1 score. However, properly dealing with such an imbalance is not a simple task, as discussed next. Finally, as the sentence length was very similar across tasks and classes, this factor did not affect the results, that is, we could not infer any significant relationship between the mean number of words per sentence and the Macro-F1 scores of the models.

**Figure 3:**
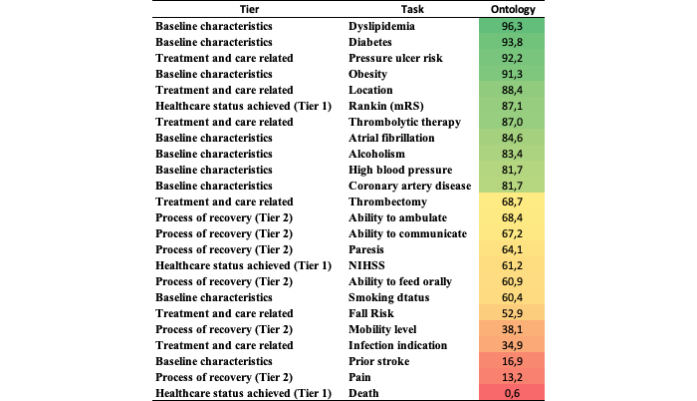
Effectiveness results for the ontology-based model. mRS: Modified Rankin Score; NIHSS: National Institutes of Health Stroke Scale.

Figure 3 provides information regarding the effectiveness of the OWL classifier. In general, the OWL effectiveness is similar to that of the best ML models, with 11 tasks having a Macro-F1 score higher than 80%. The most interesting issue is that most of the best-performing tasks by OWL *do not coincide* with the best ones produced by the ML models in Figure 2. For instance, the OWL classifier performed very well on the patient's baseline characteristics tasks, such as NIHSS and mRS scale, precisely the ones in which the ML models performed poorly. Overall, the OWL strategy was more robust in the tasks in which the ML models suffered from a scarcity of examples and high imbalance. On the contrary, OWL suffered on tasks that were much more passible in interpretation and had more text representations from those for which they were built [49,80]. For instance, in the *death* task, despite good within-annotator agreement, we believe that due to a variety of clinical terms in the clinical text used to describe multiple clinical concepts, the rules initially created failed to reflect the understanding of a noninformative sentence versus a sentence that reports the vital signs of patients, which penalized the OWL model.

**Figure 4:**
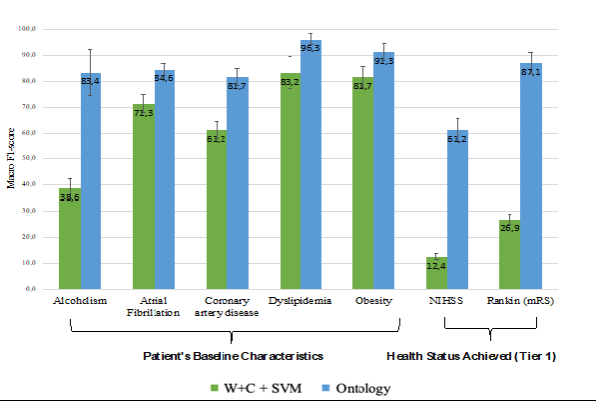
Best performed tasks in Ontology versus top-ranked model. mRS: Modified Rankin Score; NIHSS: National Institutes of Health Stroke Scale; SVM: support vector machine; W+C+SVM: word- term frequency-inverted document frequency and character- term frequency-inverted document frequency combined with support vector machine.

**Figure 5:**
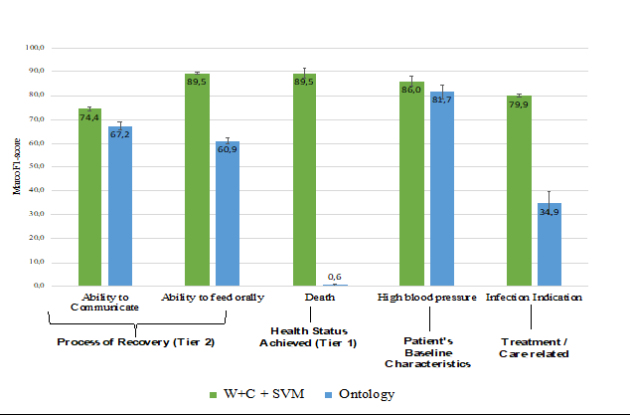
Best performed tasks in the top-ranked model versus Ontology. SVM: support vector machine; W+C: word-term frequency-inverted document frequency and character-term frequency-inverted document frequency.

A direct comparison between OWL and the best ML method is presented in Figures 4 and 5, in which Figure 4 represents the tasks in which OWL performed better than the best ML model for the same tasks and Figure 5 represents the tasks with higher F1 scores in the ML model against OWL. SVM+W+C has a considerable advantage over the other ML strategies, as the strategy of choice to be compared in the vast majority of cases. The best tasks performed by the best model in each case, either SVM+W+C or OWL, do not coincide. Indeed, there is a potential complementarity between ML and alternatives.

### Effect of Class Imbalance on the Results—Undersampling

As we have discussed, all our tasks are extremely skewed, in the sense that the NI (noninformed; majority) class dominates over the other (minority) classes, where the useful information really lies. This imbalance occurs in a proportion that can achieve 1:1000 examples in the minority class to the majority class for some tasks.

This imbalance may cause bias in the training data set influencing some of the experimented ML algorithms toward giving priority to NI class, ultimately undermining the classification of the minority classes on which predictions are most important. One approach to addressing the problem of class imbalance is to randomly resample the training data set. A simple, yet effective approach to deal with the problem is to randomly delete examples from the majority class, a technique known as random undersampling [81].

**Figure 6:**
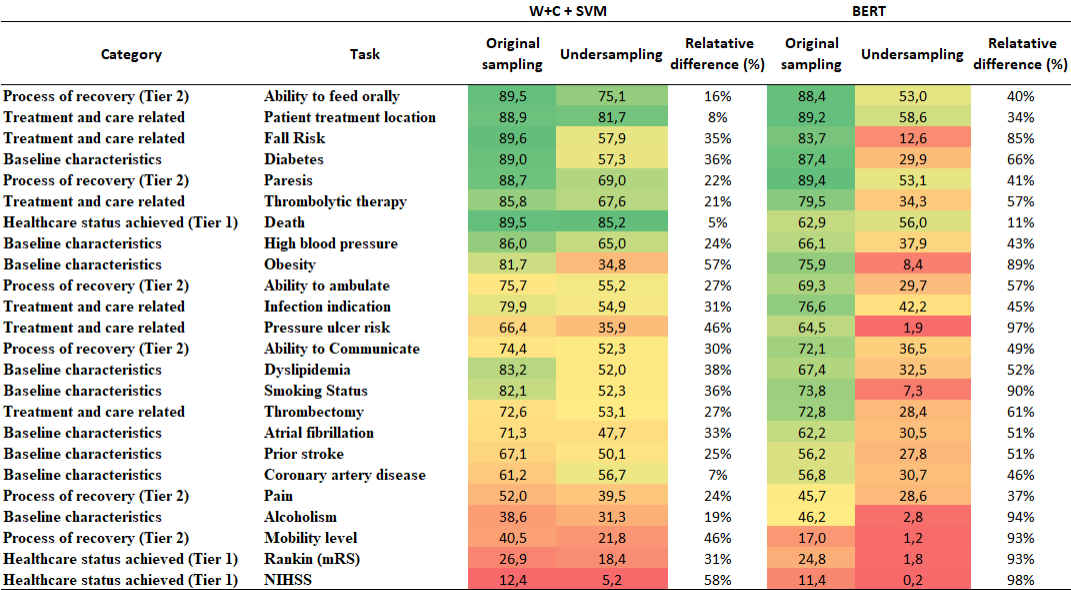
Results of Macro-F1 score in the undersampling sample, expressed by percentage. mRS: Modified Rankin Score; NIHSS: National Institutes of Health Stroke Scale; SVM: support vector machine; W+C: word- term frequency-inverted document frequency and character- term frequency-inverted document frequency.

The results of this experiment are shown in Figure 6, which compares the performance of the classifiers in scenarios with and without undersampling. For the sake of space, we only show the results for the best nonneural (W+C+SVM) and neural (BERT) classifiers, but the results are similar for all tested classifiers (Multimedia Appendix 7).

As it can been seen, the undersampling process caused major losses in both classifiers. Such losses occurred across all tasks, varying from 5% of Macro-F1 score reduction (death) to 58% (NIHSS) for W+C+SVM, and 11% (death) to 98% (NIHSS) of Macro-F1 effectiveness loss in BERT. The largest losses for the neural method were expected, as this type of classifier is more sensitive to the amount of training. However, to a certain degree, all the classifiers suffered major losses after the undersampling process. These results may be attributed to the largest difference in class distribution between training and testing and the inevitable loss of information that comes after the removal of training instances after undersampling.

These phenomena can be better seen when we look at the individual values of F1, precision, and recall of the classes of the tasks. Table 3 shows an example of the tasks of infection indication, thrombolytic therapy, and ability to communicate with the W+C+SVM classifier. As we can see, all classes have a reduced F1 in the undersampling scenario. This is mainly due to a large reduction in the precision of the classes. This happens because W+C+SVM misclassifies NI instances as belonging to some of the relevant classes. As the classifier is obliged to categorize a sentence in one of the existing classes, the lack of information about the fact that a sentence does not have useful information for assigning the sentence in one of the classes of interest confounds the classifier. In other words, the negative information about the NI (eg, frequent words in NI sentences that help to characterize this class but that are also shared by some non-NI instances, and whose frequency was altered by the undersampling) is in fact useful information for avoiding false positives, which may cause many problems in a real scenario, including false alarms, waste of resources, and distrust of the automatic methods.

**Table 3:** Comparison of undersampling and original sampling in terms of precision, recall, and Macro-F1 score (W+C+SVM model).

Class		Undersampling				Original sampling		
		Precision	Recall	F1 (%)^a^	Precision		Recall	F1 (%)^a^
**Infection indicative**								
	−1	1	0.96	98	0.99		1	99
	0	0.39	0.89	54	0.88		0.75	81
	1	0.28	0.82	42	0.68		0.53	59
**Thrombolytic therapy**								
	−1	1	0.98	99	1		1	100
	0	0.32	0.62	42	0.69		0.52	59
	1	0.31	0.91	47	0.89		0.91	90
**Ability to communicate**								
	−1	1	0.96	98	0.99		1	100
	0	0.34	0.63	44	0.9		0.26	40
	1	0.35	0.81	49	0.76		0.64	69
	2	0.32	0.93	48	0.82		0.8	81

^a^Macro-F1 score (W+C+SVM model).

### Feature Importance

For the tasks presented in Textbox 1 (alcoholism, atrial fibrillation, coronary artery disease, dyslipidemia, obesity, NIHSS, Rankin [mRs], infection indicators, high blood pressure, death, ability to feed orally, and ability to communicate), we present the top 10 clinical features (ie, words) used in the task prediction in Textbox 1, which means the 10 features with higher contribution to task prediction. This analysis helps to better understand the divergence between approaches. It is worth noting that in the tasks in which the ML models performed better (second column), the top-ranked features were all related to the semantics of the task. For instance, considering the *death* task as an example, the ML model was able to identify important features for the task, which produced a higher information gain than the OWL model. Indeed, for *death*, only three features of the 10 most relevant explicitly use the word *death*, but most features are somewhat related to this outcome. This finding suggests data quality issues (vocabulary coverage) that may drastically influence the effectiveness of the OWL strategy, which exploits only rules that explicitly contain the word *death* (or related ones) but no other terms. However, for the features in the first column, in which the OWL models were better, there were still features with considerable contributions that were not directly related to the information sought. For example, to mention the NIHSS task, rule-based knowledge models built alongside clinical domain vocabulary specialists may be the best alternative.

**Top 10 clinical indicators for task prediction models and feature importance. In parenthesis, the translation to English language is indicated, where there may be misspellings in the original writing that are also indicated.**

**Alcoholism**

- etilismo (alcoholism)
- etilista (alcoholic)
- fumo (smoke)
- históira (story with misspelling in the original)
- álcool (alcohol)
- cart
- osteoartrose (osteoarthritis)
- ttu (short for transurethral resection of the prostate)
- tabagismo (smoking)
- cesária (cesarean)

**Atrial fibrillation**

- fa (short for atrial fibrillation)
- comorbidades (comorbidities)
- acfa (short for atrial fibrillation)
- paroxística (paroxysmal)
- has (short for high blood pressure)
- anticoagulado (anticoagulated)
- depressão (depression)
- indeterminado (indeterminate)
- digoxina (digoxin)
- institucionalizada (institutionalized)

**Coronary artery disease**

- cardiopatia (heart disease)
- isquêmica (ischemic)
- actp (short for percutaneous transluminal coronary angioplasty)
- dp
- crm (short for myocardial revascularization surgery)
- iam (short for acute myocardial infarction)
- 2014
- infarto (short for acute myocardial infarction)
- mm
- sf

**Dyslipidemia**

- dislipidemia (dyslipidemia)
- comorbidades (comorbidities)
- 1hora
- cesária (cesarean)
- morbidades (morbidities)
- puerpera (puerperal)
- has (short for high blood pressure)
- fêmur (fêmur)
- tep
- previas (previous)

**Obesity**

- BMI (short for body mass index)
- obesidade (obesity)
- m²
- 1994
- lipschitz
- eutrofia
- altura (height)
- peso (weight)
- estatura (stature)
- obesa (obese)

**National Institutes of Health Stroke Scale**

- nihss
- súbito (sudden)
- asistolia (asystolia)
- sens
- territ
- suboclusiva (subocclusive)
- perg
- mecania (mecanic with mispelling in the original)
- severo (severe)
- visto (seen)

**Ability to communicate**

- afasia (afasia)
- comunicativa (talkative)
- disartria (dysarthria)
- comunicativo (talkative)
- colóquio (colloquium)
- verbalizando (verbalizing)
- alerta (alert)
- verbaliza (verbalizes)
- expressão (expression)
- hemiparesia (hemiparesis)

**Ability to feed orally**

- vo (short for orally)
- sne (short for nasoenteral probe)
- dieta (diet)
- pastosa (pasty)
- gastrostomia (gastrostomy)
- enteral (enteral)
- aceitação (acceptance)
- semi (semi)
- exclusiva (exclusive)
- polimérica (polymeric diet)

**Death**

- óbito (death)
- constato (i’ve verified)
- leito (bed)
- ar (air)
- estável (stable)
- ambiente (environment or room)
- no
- doação (donation)
- obito (death with misspelling in the original)
- óbito (death with misspelling in the original)

**High blood pressure**

- has (short for high blood pressure)
- dm (short for diabetes)
- dislipidemia (dyslipidemia)
- dm2 (short for diabetes type 2)
- comorbidades (comorbidities)
- fa (short for atrial fibrillation)
- artrite (arthritis)
- definitivo (definitive)
- reumatoide (rheumatoid)
- demencial (dementia)

**Infection indication**

- afebril (afebrile)
- flogísticos (phlogistic)
- sinais (signs)
- cefuroxima (cefuroxime)
- inserção (insertion)
- tax
- klebsiella (klebsiella)
- d0 (short for day 0)
- atb (short for antibiotics)
- azitromicina (azithromycin)

**Modified Rankin Score**

- rankin
- mrankin
- demência (dementia)
- caminha (walks)
- corversa (talks)
- alimenta (feed)
- alzheimer
- aparentes (apparent)
- comer (eat)
- mrk (mrs with misspelling in the original)

## Discussion

### Principal Findings

The study intended to recognize the path and opportunities that may be advanced in terms of the technological capacity to support the outcome measurement process for the stroke care pathway. Real-world sentences from ischemic stroke EMRs were used to develop automatic models using ML and NLP techniques. It was possible to identify that SVM+W+C and SVM+BoW were the most effective models to be used to classify characteristics of a patient and process of care based on the extraction of Brazilian-Portuguese free-text data from the EMRs of patients. Ontological rules were also effective in this task, and perhaps even more importantly, most of the best-performing tasks with the OWL and ML models did not coincide. This outcome opens up the opportunity to exploit such complementarities to improve the coverage of tasks when implementing a real solution for outcome management or even to improve the individual effectiveness of each alternative by means of ensemble techniques such as stacking [82].

One of the good practices that the literature has demonstrated to increase the success of ML algorithms applied to health care is the inclusion of a clinical background in the annotation process [83]. The availability of training data is critical in obtaining good results, thus indicating that variations in clinical terms found in the clinical text could be specific to the type and source of clinical notes that may not have been captured in an available resource. The results from our feature importance analysis are consistent with other study results [21,68,76,83-85] concerning many clinical terms applied to multiple clinical concepts, although there are specific patterns based on semantic types that can help. In general, it is difficult to determine the correct concept when a clinical term normalizes to multiple concepts, and this issue can penalize the effectiveness of the model [86,87].

Our effectiveness results agree with the literature [83,88], in which a Macro-F1 score >80% is considered a successful extraction of medical records. Even though there is still a need to cover more tasks related to ICHOM patient-reported outcome measures [3,74,76,85], we hypothesized that these tasks comprise a feeling state, and the lack of normalization of data contained in EMRs may explain the fact that these task categories did not perform very well [70,89]. Medical records related to baseline characteristics and care processes typically contain much more structured data (eg, numerical values for tasks) than medical patient-reported outcomes, which focus more on unstructured data [83,90]. This issue has been explored in previous studies on EMR-based clinical quality measures [22,82], in which it is suggested that these kinds of data (for baseline characteristics and care-related processes) have the potential to be scaled in other clinical conditions, such as cardiovascular and endocrine conditions [83].

Previous studies have found various advantages of EMR compared with traditional paper records [91]. However, as reported by Ausserhofer et al [12], care workers do not find them useful for guaranteeing safe care and treatment because of the difficulty of tracking clinical and quality measures. The same authors have discussed the importance of having IT capability to track care workers’ documentation while increasing safety and quality of care. They emphasized that this approach is important for addressing EMR data collection issues that have been historically extracted via manual review by clinical experts, leading to scalability and cost issues [83,85,90]. In our study, it was possible to demonstrate that for the stroke care pathway, the use of ML models to measure clinical outcomes remains a challenge, but the technology has the potential to support the extraction of relevant patient characteristics and care-process information.

Despite the challenges regarding the accuracy of the outcome measures, promising approaches regarding baseline characteristics and care-related process data have been achieved. This may be the first step toward unlocking the full potential of EMR data [83]. The usefulness of having baseline characteristics tracked is to assist disease prevalence studies and identify opportunities to guide political decisions about the public health sector [13,92,93], automatize eligibility of patients for clinical research [84], and feed risk assessment tools [94]. On the contrary, care-related process metrics boost the opportunity to improve decision-making with new technologies, maintain the effectiveness of treatments, and encourage alternative remuneration models [17,92,95].

The next step would be to invest in the automation of tasks at the patient level that support the control of the progression of patients in real-time during stroke episodes. In a similar manner, it would be useful to identify opportunities to improve the EMR data quality, such as the implementation of quality software with dynamic autocompletes with normalized terms register. The use of NLP for quality measures also adds to the capture of large amounts of clinical data from EMRs [82]. The products of NLP and mixed methods pipelines could potentially impact a number of clinical areas and could facilitate appropriate care by feeding hospital outcome indicators and data to support epidemiological studies or value-based programs [82].

### Limitations

This study had several limitations. For clinical NLP method development to advance further globally and to become an integral part of clinical outcome research or have a natural place in clinical practice, there are still challenges ahead. Our work is based on the EMR of a single center, with a limited number of annotated patients. Thus, further work is needed to test this approach in EMRs from different centers with different patients, who may use different languages for clinical documentation. We have no access to data from exams or hospital indicators, which is the reason why our infection identification, for example, was based on any report of antibiotic use, typical symptoms of infection, or tests described. We were unable to find data samples that included all the risk factors that were discovered in the literature. It would be worth conducting a future study with a larger and different data set with more features to examine whether the findings of this research are still valid. Finally, the design focused on sentences can be significantly influenced by the NI data volume—if a patient smokes, this will probably be reflected in just one sentence, maybe two, and for all of the others, you will have NI. One possible approach would be to use hierarchy models to first classify whether a sentence is relevant and then evolve to classification algorithms to predict classes. Then, the entire record can inform the prediction of the outcome of patients, instead of saying whether a specific sentence indicates a task.

Regarding the undersampling experiment, more intelligent strategies such as choosing the *most positive of the negative samples* or Tomek links [81] should be tested for better effectiveness. We leave this for future work and suggest practical purposes to maintain the original distribution, whereas more effective strategies are not further studied.

### Conclusions

This study is innovative in that it considered many and diverse types of automatic classifiers (neural, nonneural, and ontological) using a large real-world data set containing thousands of textual sentences from real-world EMRs and a large number of tasks (n=24) with multiple classes using Brazilian-Portuguese unstructured free-text EMR databases. The effectiveness of these models demonstrated a better result when used to classify care processes and patient characteristics than patient-reported outcomes, which suggests that advances in intelligence in informational technology for clinical outcomes are still a gap in the scalability of outcome measurements in health care. Future research should explore the development of mixed methods to increase task effectiveness. Advances in IT capacity have proved to be essential for the scalability and agility of the ability to measure health outcomes and how it reflects on its external validation to support health real-time quality measurement indicators.

## Appendix

Multimedia Appendix 1 Example of an evolution on the electronic medical record.

Multimedia Appendix 2 Example of the annotation process.

Multimedia Appendix 3 Data set characteristics.

Multimedia Appendix 4 Details of the automatic text classification methods.

Multimedia Appendix 5 Experimental procedure.

Multimedia Appendix 6 Experimental protocol details—specific parameter tuning.

Multimedia Appendix 7 Results of F1 score from the random undersampling experiment. BERT: bidirectional encoder representation from transformers; BoW: Bag-of-Words; KNN: K-nearest neighbor; mRS: Modified Rankin Score; NIHSS: National Institutes of Health Stroke Scale; SVM: support vector machine; TFIDF: term frequency-inverted document frequency; W+C: word- term frequency-inverted document frequency and character- term frequency-inverted document frequency.

## Abbreviations

BERT: bidirectional encoder representation from transformers
BoW: Bag-of-Words
CNN: convolutional neural network
EMR: electronic medical record
IT: information technology
KNN: K-nearest neighbor
ML: machine learning
NIHSS: National Institutes of Health Stroke Scale
NLP: natural language processing
OWL: ontology web language
SVM: support vector machine
TFIDF: term frequency-inverted document frequency
