# Supplementary material for: Stroke Outcome Measurements From Electronic Medical Records: Cross-sectional Study on the Effectiveness of Neural and Nonneural Classifiers
Source: JMIR Med Inform. 2021 Nov 1;9(11):e29120. doi: 10.2196/29120 (PMC8593798; doi:10.2196/29120)
Supplement: Multimedia Appendix 1 [file medinform_v9i11e29120_app1.docx]

**Multimedia Appendix 1 [Table S1]**

**Tables S1.** Example of an evolution on Electronic Medical Record

| Example 1 |
| --- |
| Admission note: emergency nursing  Patient XXX, age XXX, interned for ischemic stroke. Patient with fall from his own height, and loss of strength in the lower limbs. Severe headache.  You are not aware of allergies.  #HBP;  Ischemic stroke;  #Osteoporosis.  S- Patient with no complaints at the moment, including pain.  O- Patient lying on the bed, LOC and quiet. Ventilates in room air. In NPO. Diuresis present in bedpan. Diaper for comfort. Peripheral venous catheter in saline MSE. Skin intact. Walks with home help. No family member present at the moment.  C- Observe sensory. Observe skin integrity. Observe phlogistic signs when inserting a venous catheter. Communicate changes. Nursing care.  I transfer the patient to an adult ICU bed box 65. |
| Example 2 |
| Neurological Physiotherapy - 09:50  Setting: Patient meeting in bed, accompanied, alert, plegic in MSE, paretic MIE.  Ventilatory Condition: Ventilating in room air, maintains a good ventilation pattern. AP: MV decreased without RA  Functionality: Changed Origin of limitation: Neurological  Mobility Level: 8  Conducts: Passive mobilizations of MSE and passive of MIE.  Light resisted assets of MSD and MID. Bedside sedation, reinforcement of cervical straightening training, poor performance.  Pivot for armchair and MsIs / MsSs stretches.  Plan: to stimulate active movements, to train to roll, to train the trunk, to try orthostasis when in conditions.  Notes: Stay in the chair as long as possible.  It is in the armchair, stable, accompanied. |

The examples were literally translated from Brazilian-Portuguese and it may be different in clinical reporting pattern.
