# Supplementary material for: Stroke Outcome Measurements From Electronic Medical Records: Cross-sectional Study on the Effectiveness of Neural and Nonneural Classifiers
Source: JMIR Med Inform. 2021 Nov 1;9(11):e29120. doi: 10.2196/29120 (PMC8593798; doi:10.2196/29120)
Supplement: Multimedia Appendix 2 [file medinform_v9i11e29120_app2.docx]

**Multimedia Appendix 2 [Table S2]**

**Table S2**. Example of the annotation process.

| Task | Hospital Location | Thrombolytic therapy | Dyslipidemia | High blood pressure | Diabetes | NIHSS | Mobility Level |
| --- | --- | --- | --- | --- | --- | --- | --- |
| Sentence / Classes | NI,1-2-3-4 | NI,0,1 | NI,0,1 | NI,0,1 | NI,0,1 | NIHSS score | 1-15 score |
| Admission note: emergency nursing, | 1 | - | - | - | - | - | - |
| Patient XXX, age XXX, interned for ischemic stroke., | - | - | - | - | - | - | - |
| Patient with fall from his own height, and loss of strength in the lower limbs., | - | - | - | - | - | - | - |
| Severe headache., | - | - | - | - | - | - | - |
| You are not aware of allergies., | - | - | - | - | - | - | - |
| #HAS ;, | - | - | - | 1 | - | - | - |
| # Dyslipidemia | - | - | 1 | - | - | - | - |
| - thrombolysis (NIHSS: 2). | - | 1 | - | - | - | 2 | - |
| S- Patient without complaints, | - | - | - | - | - | - | - |
| at the moment, including pain., | - | - | - | - | - | - | - |
| I transfer the patient to the ICU bed, | 3 | - | - | - | - | - | - |
|  | - | - | - | - | - | - | - |
| Neurological Physiotherapy -, | - | - | - | - | - | - | - |
| 09:50 Setting: Patient encounter, | - | - | - | - | - | - | - |
| in bed, accompanied, alert, plegic in MSE, paretic MIE., | - | - | - | - | - | - | - |
| Ventilatory Condition :, | - | - | - | - | - | - | - |
| Ventilating in room air, maintains a good ventilation pattern., | - | - | - | - | - | - | - |
| AP: MV decreased without RA Functionality: Changed Origin of limitation: Neurological Mobility Level: 8 Conducts: Passive mobilizations of MSE and passive of MIE., | - | - | - | - | - | - | 8 |
| Light resisted assets of MSD and MID., | - | - | - | - | - | - | - |
| Photography, | - | - | - | - | - | - | - |
| at bedside, strengthening cervical straightening training, poor performance., | - | - | - | - | - | - | - |
| Pivot for armchair and MsIs stretches /, | - | - | - | - | - | - | - |
| MsSs., | - | - | - | - | - | - | - |
| Plan: to stimulate active movements ,, | - | - | - | - | - | - | - |
| train to roll, train the trunk, try orthostasis when in conditions., | - | - | - | - | - | - | - |
| Comments:, | - | - | - | - | - | - | - |
| Stay in the armchair as long as possible., | - | - | - | - | - | - | - |
| Stay in the armchair, stable, accompanied. | - | - | - | - | - | - | - |

NI, non-informative sentence represented as hyphen (-). Example, a task referring to a patient's "thrombolytic therapy" might contain three answers (classes: "no delta," "yes," "non-informative"). Therefore, the annotation process consisted of indicating which answer for the task "thrombolytic therapy" each sentence inferred. One sentence could indicate the patient had not realized thrombolytic therapy during the inpatient period, or even the sentence could be a non-informative (NI) sentence that means the sentence is not related to the patient's thrombolytic therapy status
