# Supplementary material for: Stroke Outcome Measurements From Electronic Medical Records: Cross-sectional Study on the Effectiveness of Neural and Nonneural Classifiers
Source: JMIR Med Inform. 2021 Nov 1;9(11):e29120. doi: 10.2196/29120 (PMC8593798; doi:10.2196/29120)
Supplement: Multimedia Appendix 3 [file medinform_v9i11e29120_app3.docx]

**Multimedia Appendix: [Table S3]**

**Table S3** – Dataset Characteristics

|  |  |  |  | Class distribution per task | |  | Sentence Distribution | | |  |  |  |
| --- | --- | --- | --- | --- | --- | --- | --- | --- | --- | --- | --- | --- |
| Task | Size (clinical narrative) | Size (sentences) | # Classes | Minor Class (sentences) | Major Class (sentences) | Median (words) | Mean (words) | Min (words) | Max (words) | Distinct Patients (n) | Skewness |  |
| Ability to ambulate | 1,558 | 44,206 | 4 | 106 | 43,479 | 15.2 | 8.4 | 1 | 405 | 188 | Extremely Umbalanced |  |
| Ability to feed orally | 1,558 | 44,206 | 3 | 689 | 42,657 | 9.2 | 6,7 | 1 | 405 | 188 | Extremely Umbalanced |  |
| Alcoholism | 1,558 | 44,206 | 4 | 22 | 44,097 | 20.4 | 16.1 | 1 | 405 | 188 | Extremely Umbalanced |  |
| Atrial Fibrillation | 1,558 | 44,206 | 3 | 14 | 43,914 | 10.4 | 9.8 | 1 | 405 | 188 | Extremely Umbalanced |  |
| Coronary artery disease | 1,558 | 44,206 | 3 | 24 | 43,890 | 13 | 8.3 | 1 | 405 | 188 | Extremely Umbalanced |  |
| Death | 1,558 | 44,206 | 3 | 9 | 41,871 | 6 | 8.12 | 1 | 405 | 188 | Extremely Umbalanced |  |
| Diabetes | 1,558 | 44,206 | 3 | 39 | 43,859 | 7.8 | 6.7 | 1 | 405 | 188 | Extremely Umbalanced |  |
| Dyslipidemia | 1,558 | 44,206 | 3 | 8 | 44,063 | 7.9 | 6 | 1 | 405 | 188 | Extremely Umbalanced |  |
| Fall Risk | 1,558 | 44,206 | 4 | 115 | 43,763 | 8 | 12.5 | 1 | 405 | 188 | Extremely Umbalanced |  |
| High blood pressure | 1,558 | 44,206 | 3 | 25 | 43,624 | 7.8 | 7 | 1 | 405 | 188 | Extremely Umbalanced |  |
| Infection Indication | 1,558 | 44,206 | 4 | 362 | 43,234 | 11.5 | 8.3 | 1 | 405 | 188 | Extremely Umbalanced |  |
| Location | 1,558 | 44,206 | 4 | 109 | 42,694 | 13 | 13.9 | 1 | 405 | 188 | Extremely Umbalanced |  |
| Mobility level | 1,558 | 44,206 | 16 | 3 | 43,560 | 17.9 | 11.6 | 1 | 405 | 188 | Extremely Umbalanced |  |
| NIHSS | 1,558 | 44,206 | 42 | 2 | 43,903 | 5 | 8 | 1 | 405 | 188 | Extremely Umbalanced |  |
| Obesity | 1,558 | 44,206 | 3 | 27 | 44,121 | 11.4 | 8,5 | 1 | 405 | 188 | Extremely Umbalanced |  |
| Pain | 1,558 | 44,206 | 4 | 23 | 43,581 | 9.9 | 7.7 | 1 | 405 | 188 | Extremely Umbalanced |  |
| Paresis | 1,558 | 44,206 | 2 | 505 | 43,701 | 11.1 | 8.5 | 1 | 405 | 188 | Extremely Umbalanced |  |
| Pressure Ulcer Risk | 1,558 | 44,206 | 4 | 59 | 43,949 | 11.1 | 9.2 | 1 | 405 | 188 | Extremely Umbalanced |  |
| Prior stroke | 1,558 | 44,206 | 3 | 29 | 43,969 | 10.5 | 9.7 | 1 | 405 | 188 | Extremely Umbalanced |  |
| Rankin (mRS) | 1,558 | 44,206 | 7 | 11 | 44,019 | 10.2 | 8.5 | 1 | 405 | 188 | Extremely Umbalanced |  |
| Smoking Status | 1,558 | 44,206 | 4 | 32 | 43,923 | 13.2 | 16.2 | 1 | 405 | 188 | Extremely Umbalanced |  |
| Thrombectomy | 1,558 | 44,206 | 3 | 25 | 43,971 | 16.9 | 11 | 1 | 405 | 188 | Extremely Umbalanced |  |
| Thrombolytic therapy | 1,558 | 44,206 | 3 | 107 | 43,708 | 13 | 14.4 | 1 | 405 | 188 | Extremely Umbalanced |  |

Minor class, less expressed class in number of sentence; Major class, most present class in number of sentences; Mean, average words per sentence per class; Median, median words per sentence per class. Example: "thrombolytic therapy" task has three classes (classes: "no delta," "yes," "non-informative"), with the minor one containing 107 sentences and the majority one having 43,708 sentences. This is expected since, for most tasks, the vast majority of the sentences are not related to the task at all. We chose to maintain this imbalance as in the original dataset in order to simulate an environment as close to reality as possible. The same table informs, for each task, the sentence distribution in words. For the same example of thrombolytic therapy, the median number of words per annotated sentence is 13, and the mean is 14.4.
