# Supplementary material for: Stroke Outcome Measurements From Electronic Medical Records: Cross-sectional Study on the Effectiveness of Neural and Nonneural Classifiers
Source: JMIR Med Inform. 2021 Nov 1;9(11):e29120. doi: 10.2196/29120 (PMC8593798; doi:10.2196/29120)
Supplement: Multimedia Appendix 4 [file medinform_v9i11e29120_app4.docx]

**Multimedia Appendix 4: [Automatic Text Classification Methods details]**

**Automatic Text Classification Methods details.**

More details about the 2P2 methods are given below:

The first category of methods consists of approaches whose document representation is intrinsically independent of the class prediction phase's classification method. In other words, the classifier used to predict the class of the documents is not used in the phase of construction of the document representation. In terms of text representations, we compare traditional term-weighting alternatives, weighted by word and character, and recent representations based on meta-features that have obtained state-of-the-art effectiveness in some of the experimented datasets.

**Term Frequency – Inverse Document Frequency (TF-IDF)** [50, 51] aims to reflect the importance of a word in a document, given a corpus. Based on a BoW model, a document is converted into a multi-hot encoding of words in it, based on vocabulary constructed from the corpus. Then, words are given weights based on their importance for each document. A word's importance increases proportionally to the number of times it appears in that document (term frequency) and inversely proportional to the total of documents that contain it (inverse document frequency). There may be variations of the TF-IDF. The most common in this objective are Word TF-IDF, which corresponds to analyze the terms present in the document, and Char TF-IDF analyzes a character sequence defined here between the range of 2 to 6 characters. Concerning Word TF-IDF and Char TF-IDF, Char can better capture terms with typos because it analyzes part of a term defined in the interval. For example, if we analyze the term 'smoker' and 'smokre", he considers that both terms are similar for a defined range of the first four terms. It is common to use the concatenation of the vectors of both representations, Word TF-IDF and Char TF-IDF, in a single representation. This vector is used as input for a classifier, which will be presented in the next section. The purpose of this combination is to enhance the benefits of both representations [39].

**Bag of Words (BoW)** is a reduced and simplified representation of a document text of selected parts of the text, based on specific criteria, such as word frequency. The BoW technique is used in several domains, such as computer vision, PLN, document classification, and information retrieval by machine learning [42].

**Meta-features (MF)** it scores are obtained by functions that relate textual data to categories. In this work, MFs were explored based on the distance functions between text examples that have recently obtained the most effective results in several text categorization problems. The rationale behind these MF is the assumption that if the distances between a text example and the closest neighbors belonging to the "c" category (and its corresponding centroid) are small, then the example probably belongs to "c." Such distance scores are additional evidence, which, when concatenated to the original representation, can assist in the construction of classification models in collections with a large number of labeled examples [37].

The chosen classification algorithms are detailed below:

**Support Vector Machine (SVM)** is a popular and successful technique that employs a discriminative classifier for document categorization [52, 60, 61]. SVM is among the most used methods in the health area [3, 62].

**Naive Bayes Classifier (NBC)** [43, 53] is one of the primary techniques used in information retrieval. It is still widely used because it is computationally cheap and requires a meager amount of memory. This technique is a generative model, which is the most traditional text categorization method. This algorithm is also used as a reference (or baseline benchmark) for many surveys to highlight news and contributions [54].

**K-Nearest Neighbor (KNN)** [44] is a technique that performs well on fundamental recognition problems. Given a test document *d*, the KNN algorithm finds the k neighbors closest to *d* among all the documents in the training set and scores the category candidates based on k neighbors' class. The similarity of *d* and each neighbor's is used to compute the score of the category of the neighbor documents. After ranking the score values, the algorithm assigns the candidate document d to the class with the highest score.

**Random Forest (RF)** [41, 63] is another well studied algorithm concerning document categorization. The algorithm exploits the use of many (hundreds of) decisions trees in a voting scheme and a bagging scheme for training the trees to exploit data randomness. In recent text classification benchmarks, it has produced competitive effectiveness [35, 39, 55].

More details about the E2E methods are given below:

**Convolutional Neural Network (CNN)** [56] implemented in this work consists of an embedding layer loaded with Word2Vec word vectors, followed by a single one-dimensional convolutional layer and Batch Normalization. Deep learning approaches such as CNN have achieved superior results compared to previous machine learning algorithms in image classification, some natural language processing tasks, and facial recognition, among others [57].

**Bidirectional Encoder Representations from Transformers (BERT)** [58] is a pre-trained end-to-end deep learning method with a corpus of 3.3 billion words, including Books Corpus (800 million words) and Wikipedia in English (2.5 billion words). BERT uses a multi-layer bidirectional Transformer encoder with self-attention in both directions. In short, it predicts missing words from a sentence. BERT redefined state of the art for natural language processing tasks. This deep learning algorithms’ success depends on their ability to model complex and non-linear relationships within the data [59].

**Rule based (Ontology):**

Ontologies are specifications, explicit and formal, of the conceptualization of a domain of interest. A formal specification is understood by a computer that with this understanding can deduce about the concepts [45]. Usually, a vocabulary of terms and the specifications of their meanings are included and definitions and relations of how the concepts are interrelated, imposing a structure on a domain and limiting the possible interpretations of the terms [59]. With a textual search algorithm, the terms in the sentences are identified, and a class is defined in the ontology for a set of terms and their relations. Based on the terms and relationships, the inference process is performed where it deduces which variables are represented in a given sentence.
