# Supplementary figures and images for: Stroke Outcome Measurements From Electronic Medical Records: Cross-sectional Study on the Effectiveness of Neural and Nonneural Classifiers
Source: JMIR Med Inform. 2021 Nov 1;9(11):e29120. doi: 10.2196/29120 (PMC8593798; doi:10.2196/29120)

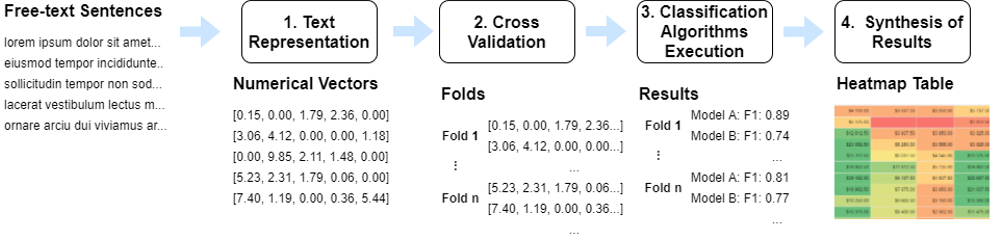

Supplement: Multimedia Appendix 5 [file medinform_v9i11e29120_app5.png]

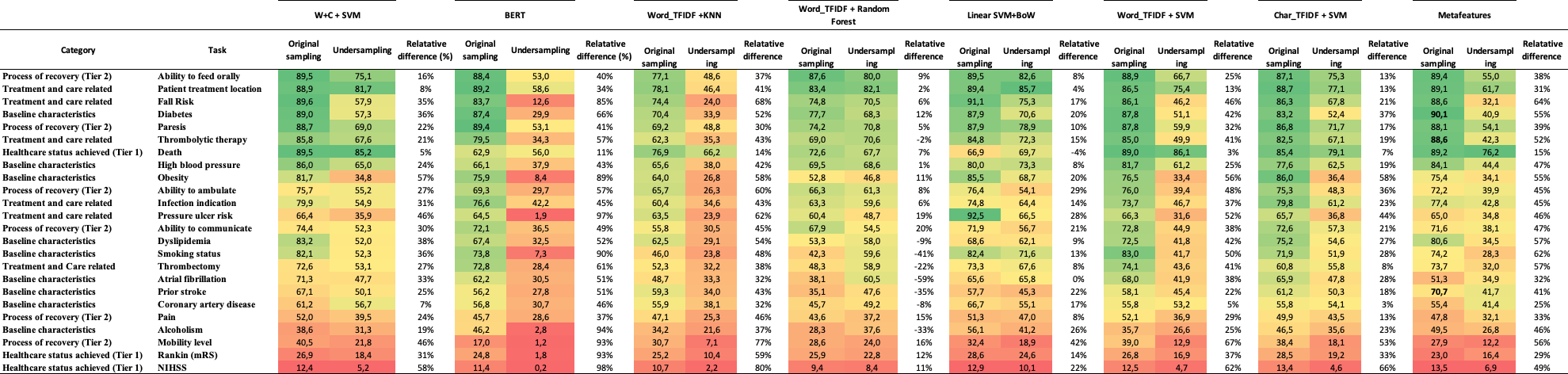

Supplement: Multimedia Appendix 7 [file medinform_v9i11e29120_app7.png]
