# Supplementary material for: Stroke Outcome Measurements From Electronic Medical Records: Cross-sectional Study on the Effectiveness of Neural and Nonneural Classifiers
Source: JMIR Med Inform. 2021 Nov 1;9(11):e29120. doi: 10.2196/29120 (PMC8593798; doi:10.2196/29120)
Supplement: Multimedia Appendix 6 [file medinform_v9i11e29120_app6.docx]

### Multimedia Appendix 6 [Experimental Protocol details -Specific Parameter Tuning]

### Experimental Protocol details -Specific Parameter Tuning

We perform different and suitable strategies to define each model's parameters since there are considerable differences in functioning among them. We list below the configurations used in each model in order to assist in the reproducibility of the results obtained in this study:

**SVM and W+C:** The SVM kernel used was Linear. We evaluated the C parameter varying among [10 ^(−4)^ , 10 ^(−3)^ , 10 ^( −2)^ , 10 ^(−1)^ , 10^0^ , 10^1^ , 10^2^, 10^3^ , 10^4^] and using a grid search strategy with folded cross-validation under each fold's training data. For Word-TFIDF, we use unigram (n=1) and bigram (n=2). For the Char-TFIDF representation it uses the range between 2-6 characters.

**SVM and BoW:** The SVM kernel used was Linear. We tune the parameter C considering the values from 0.001 to 1000 and using a grid search strategy with folded cross-validation under each fold's training data.

**Metafeatures:** The SVM kernel used was Linear. We evaluated the n_neighbors parameter among [10, 20, 30, 40, 50] and the C parameter varying among [10^(−4)^ , 10 ^(−3)^ , 10^( −2)^ , 10 ^(−1)^ , 10^^ 0^ , 10^^ 1^ , 10^^ 2^ , 10^^ 3^ , 10^^ 4^ ]  using a grid search strategy with folded cross-validation under each fold's training data.

**SVM + Word-TFIDF:** The SVM kernel used was Linear. We evaluated the C parameter varying among [10^(−4)^ , 10^(−3)^ , 10^( −2)^ , 10 ^(−1)^ , 10^0^ , 10 ^1^ , 10^2^ , 10^3^ , 10^4^ ] and using a grid search strategy with folded cross-validation under each fold's training data. For Word-TFIDF, we use unigram (n=1) and bigram (n=2).

**SVM + Char-TFIDF:** The SVM kernel used was Linear. We evaluated the C parameter varying among [10^(−4)^ , 10^(−3)^ , 10^( −2)^ , 10 ^(−1)^ , 10^0^ , 10 ^1^ , 10^2^ , 10^3^ , 10^4^] and using a grid search strategy with folded cross-validation under each fold's training data. For the Char-TFIDF representation it uses the range between 2-6 characters.

**KNN + Word-TFIDF:** In KNN, we evaluated the n_neighbors parameter varying among [1, 2, 4, 8, 16, 32, 64, 128] and using a grid search strategy with folded cross-validation under each fold's training data. For Word-TFIDF, we use unigram (n=1) and bigram (n=2).

**RF + Word-TFIDF:** In Random Forest, we evaluated the n_estimator parameter varying among [10, 50, 100, 200] and using a grid search strategy with folded cross-validation under each fold's training data. For Word-TFIDF, we use unigram (n=1) and bigram (n=2).

**Naive Bayes + Word-TFIDF:** In Naive Bayes we use Multinomial, optimizing the 'alpha' parameter in the interval between [[10^(−6)^ 10^(−5)^, 10^(−4)^, 10^(−3)^, 10^( −2)^, 10^(−1)^, 10^0^, 10^1^]and using a grid search strategy with folded cross-validation under each fold's training data. For Word-TFIDF, we use unigram (n=1) and bigram (n=2).

**OWL:** A list of terms and the relations between these terms and the tasks, described in the ontology

**BERT:** We used a pre-trained model (Bert Base) available in the method repository and conducted a fine-tuning on the data. We consider the meaningful input tokens up to the max sequence length (in the case 150 tokens). Besides that, we add a fully connected layer on Bert output in order to be able to use the model to perform the classification. Also, we define the batch size as 32, the initial learning rate as 5-5 and patience as 5 epochs. By definition, patient is the number of epochs with no improvement of loss on the validation set, thus, in other words, BERT only stops training when there is no change in the validation loss in 5 patience epochs.

**CNN:** CNN uses three 2D convolution layers with 512 filters, kernel sizes are 3, 4 and 5 respectively. Each convolution layer uses the standard ReLU activation function, and the embedding dimension is 100. We do not use tuning with cross-validation.
